# Dexmedetomidine attenuates inflammation and organ injury partially by upregulating Nur77 in sepsis

**DOI:** 10.1002/iid3.883

**Published:** 2023-06-21

**Authors:** Qian Zhang, Yun Huang, Chenchen Gong, Yan Tang, Jie Xiong, Difen Wang, Xu Liu

**Affiliations:** ^1^ Department of Critical Care Medicine Guizhou Medical University Guiyang Guizhou People's Republic of China; ^2^ Department of Nephrology First People's Hospital Guiyang Guizhou People's Republic of China; ^3^ Department of Thoracic and Cardiovascular Surgery The Children's Hospital of Zhejiang University School of Medicine Zhejiang People's Republic of China; ^4^ Department of Critical Care Medicine Affiliated Hospital of Guizhou Medical University Guiyang Guizhou People's Republic of China; ^5^ Department of Hematology The Affiliated Hospital of Guizhou Medical University Guiyang Guizhou People's Republic of China

**Keywords:** anti‐inflammation, dexmedetomidine, Nur77, organ injury, RAW264.7 cells

## Abstract

**Purpose:**

The aim of this study was to investigate the effect of dexmedetomidine (Dex) on inflammation and organ injury in sepsis, as well as the potential relationship between Dex and nuclear receptor 77 (Nur77).

**Methods:**

We investigated the effects of dexmedetomidine on lipopolysaccharide (LPS)‐induced inflammation in RAW264.7 cells and organ injury in the cecal ligation and puncture (CLP) mouse model. Additionally, we examined the relationship between dexmedetomidine and Nur77. The expression levels of Nur77 in RAW264.7 cells were analyzed under various types of stimulation using quantitative reverse transcription polymerase chain reaction and western blot analysis. Inflammatory cytokine levels in the cells were evaluated using enzyme‐linked immunoassay. Organ injuries were assessed by examining tissue histology and pathology of the lung, liver, and kidney.

**Results:**

Dexmedetomidine increased the expression of Nur77 and IL‐10, and downregulated inflammatory cytokines (IL‐1β and TNF‐α) in LPS‐treated RAW264.7 cells. The effect of dexmedetomidine on inhibiting inflammation in LPS‐treated RAW264.7 cells was promoted by overexpressing Nur77, while it was reversed by downregulating Nur77. Additionally, dexmedetomidine promoted the expression of Nur77 in the lung and CLP‐induced pathological changes in the lung, liver, and kidney. Activation of Nur77 with the agonist Cytosporone B (CsnB) significantly suppressed the production of IL‐1β and TNF‐α in LPS‐treated RAW264.7 cells. In contrast, knockdown of Nur77 augmented IL‐1β and TNF‐α production in LPS‐treated RAW264.7 cells.

**Conclusion:**

Dexmedetomidine can attenuate inflammation and organ injury, at least partially, via upregulating Nur77 in sepsis.

## Graphical abstract

Dexmedetomidine attenuates inflammation and organ injury by upregulating Nur77 in sepsis.

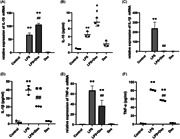

## 1 INTRODUCTION

Sepsis is a life‐threatening organ dysfunction caused by a dysregulated response of the host to infection.^1^ It is a major cause of morbidity and mortality in intensive care units worldwide and continues to present significant challenges in treatment.^2^ Macrophages are critical to homeostasis and disease, and recent research has highlighted their contribution to various processes during sepsis.^3^, ^4^ Reports suggest that studying the metabolic status of macrophages is more informative than focusing solely on their function.^4^ Several drugs have been found to alleviate sepsis by targeting macrophage‐related pathophysiological processes, including dexmedetomidine (Dex), dexamethasone, and propofol.^5^, ^6^, ^7^

The transcription factor, nuclear receptor 77 (Nur77), is an orphan nuclear receptor that belongs to the nuclear receptor 4A (NR4A) family. In addition to its transcriptional regulation, it is involved in various biological processes through non‐transcriptional regulation, such as binding to mitochondria.^8^, ^9^ Nur77 is expressed in different macrophages in response to lipopolysaccharide (LPS) stimulation, which helps regulate macrophage inflammatory response, phagocytosis, polarization, apoptosis, and mitochondrial metabolism.^10^, ^11^, ^12^, ^13^ However, the effects of Nur77 in macrophages vary by cell type and stimulus: Nur77 can either inhibit or promote macrophage inflammatory response and exhibit variable or multiple biological effects.^14^, ^15^, ^16^ Notably, recent studies have shown that Nur77 activation alleviates LPS‐induced acute lung injury (ALI), and Nur77‐deficient mice exhibit a worse disease outcome in cecal ligation and puncture (CLP).^17^ Although recent studies have emphasized that Nur77‐deficient mice are more prone to developing systemic sepsis, further studies are needed to fully understand the roles of NR4A receptors in inflammation before considering them as new targets for treating inflammatory diseases.^8^, ^18^, ^19^

Some α2 adrenoceptor agonists, such as Dex, have been shown to have anti‐inflammatory and immunosuppressive effects in addition to their sedative, analgesic, bradycardic, and hypotensive properties.^20^, ^21^ Dex has been demonstrated to decrease the mortality of sepsis.^22^, ^23^ Furthermore, sedation with dexmedetomidine has been shown to reduce inflammation in sepsis patients requiring mechanical ventilation.^24^ Dex has protective effects on the lung, liver, and kidney function as it reduces sepsis‐induced injury and apoptosis.^3^ In animal models, Dex has been reported to protect against pulmonary ischemia‐reperfusion injury, which is associated with the modulation of the miR‐21‐5p/Nr4a1 signaling pathway.^5^ However, the mechanism by which Dex attenuates inflammation and organ injury in sepsis is still unclear.

In this study, we examined whether Dex possesses anti‐inflammatory activities in in vivo and in vitro sepsis models by regulating Nur77. These findings indicated the beneficial effects and potential pharmacological mechanisms of Dex in sepsis treatment. Notably, our results confirm a potential link between Dex and Nur77 in sepsis.

## 2 MATERIAL AND METHODS

### 2.1 Cell culture and treatment

RAW264.7 cells (FuHeng) were cultured in Dulbecco's modified Eagle's medium (DMEM; Thermo Scientific) supplemented with 10% fetal bovine serum (FBS; Thermo Scientific) and 1% penicillin/streptomycin in an incubator (37°C, 5% CO_2_). The cells were treated with 10% FBS/DMEM for 24 h before administration. Concentrations of LPS, cytosporone *B* (Csn‐B), and Dex were prepared as described.^25^, ^26^, ^27^, ^28^, ^29^, ^30^ Dex preconditioning was used based on the current evidence of its potent anti‐inflammatory effect.^28^, ^29^ Both the LPS group and the Dex + LPS group were treated with 500 ng/mL LPS (Sigma) for 3 h, while the control group was not treated with LPS. Before LPS induction, the agonist group was pretreated with 10 g/mL Csn‐B (MCE) for 24 h, and the DMSO group was used as vehicle control, while the Dex + LPS and Dex groups were pretreated with 10 μM Dex (Topscience) for 30 min. For all experiments, the cells were seeded in six‐well plates at a density of 1.0 × 10^6^ cells/mL and incubated for 24 h.

### 2.2 Animal studies

C57BL/6 male mice aged 8–10 weeks and weighing 22 ± 2 g were obtained from the Laboratory Animal Center of Guizhou Medical University. C57BL/6 male mice were housed in standard cages at 25°C on a 12/12 h light–dark cycle in a clean room and supplied with food and water ad libitum. Thirty mice were randomly divided into three groups: the sham group (sham surgery, *n* = 10), septic animals (CLP group, *n* = 10), and the CLP+Dex group (CLP+Dex, *n* = 10). CLP was performed as previously described.^30^, ^31^ CLP mice were anesthetized by intraperitoneal injection with 0.2 mL/10 g 1.25% avertin anesthesia. Under sterile conditions, a 1‐cm incision was created in the lower abdominal region, and the cecum was exposed. The model was performed by ligating 50% of the cecum, puncturing once with a 21‐gauge needle, and then replacing it in the peritoneal cavity. Subsequently, the peritoneal wall and skin were closed with double sutures. Next, an intraperitoneal injection of 1 mL sterile saline (0.9%) was administered to the mice after surgery. Aside from the ligation and puncture of the cecum, all sham‐operated animals underwent the same steps. After the procedure, mice had access to water and food ad libitum. Dex (40 μg/kg) was injected into the mice by intraperitoneal injection 30 min before the operation. Three hours after the operation, mice were anesthetized with isoflurane. The liver, lung, and kidney were harvested for the detection of Nur77 expression by histopathology.

The methods were performed in accordance with the approved guidelines, and every effort was made to minimize suffering. The study was approved by the Animal Ethics Committee of Guizhou Medical University.

### 2.3 siRNA silencing

Negative control siRNA (siNC) contrast and siRNA were synthesized by GenePharma. The lipofectamine 3000 (No. GK20006) was obtained from Glpbio Technology Company. The template of siRNA for subsequent interference was determined as follows: siNur77: 5′‐GGU GAU UGG AUU GAC AAC‐ATT‐3′; siNC: 5′‐UUC UCC GAA CGU GUC ACG UTT‐3′. RAW264.7 cells were transfected with 100 pmol siNur77 or siNC in Lipofectamine 3000 according to the manufacturer's recommendation. Six hours later, the medium was replaced by DMEM + 10% FBS, cultured for an additional 72 h, and then subjected to the following treatments.

Following cell transfection with siNur77, the treatments for different cell groups were as follows: (1) siNC, transfection with siNC only; (2) siNC + Dex, siNC transfection and Dex treatment; (3) LPS + siNC, siNC transfection with LPS treatment; (4) LPS + Dex + siNC, siNC transfection with Dex and LPS treatment; (5) siNur77, transfection with siNur77 only; (6) siNur77 +  Dex, siNur77 transfection and Dex treatment; (7) LPS +  siNur77, siNur77 transfection with LPS treatment; (8) LPS + Dex + siNur77, siNur77 transfection with Dex and LPS treatment. The concentrations of Dex and LPS to be used were selected as previously described.^25^, ^26^, ^27^

### 2.4 Real‐time quantitative polymerase chain reaction (RT‐qPCR) analysis

After treatment, total RNA was prepared using an RNAprep Pure Cell/Bacteria Kit (ES) and reverse‐transcribed into first‐strand cDNA using FastKing gDNA Dispelling RT SuperMIX (Tiangen). Talent qPCR PreMix (Tiangen) was used to perform RT‐qPCR according to the manufacturer's instructions. qRT‐PCR primers used for amplification of Nur77, interleukin (IL)‐1β, IL‐10, and tumor necrosis factor (TNF)‐α were mouse Nur77 (forward primer: 5′‐CTT GAG TTC GGC AAG CCT A‐3′, reverse primer: 5′‐GGT GTC AAA CTC TCC GGT GT‐3′); mouse GAPDH primer (forward primer 5′‐GGT TGT CTC CTG CGA CTT CA‐3′, reverse primer 5′‐TGG TCC AGG GTT TCT TAC TCC‐3′); mouse IL‐1β primer (forward primer 5′‐GCA ACT GTT CCT GAA CTC AAC T‐3′, reverse primer 5′‐ATC TTT TGG GGT CCG TCA ACT‐3′); mouse IL‐10 primer (forward primer 5′‐CAG GAC TTT AAG GGT TAC TTG G‐3′, reverse primer 5′‐GGC CTT GTA GAC ACC TTG GTC‐3′); mouse TNF‐α primer (forward primer 5′‐CTC ACA CTC AGA TCA TCT TCT‐3′, reverse primer 5′‐GCT ACG ACG TGG GCT ACA G‐3′). All cytokine mRNA levels were normalized to *GAPDH* mRNA. The 2^−^^ΔΔCt^ method was used to calculate the relative changes in mRNA expression.

### 2.5 Western blot

Equal amounts of extracts (30 μg) were separated by sodium dodecyl sulfate‐polyacrylamide gel electrophoresis (10%) and transferred onto polyvinylidene fluoride (0.45 μm) membranes, followed by blocking with 5% skimmed milk at room temperature for 1 h. Subsequently, the membranes were incubated with the primary antibodies Nur77 (1:2000 dilution; Abcam) and GAPDH (1:5000 dilution; Huabio) at 4°C overnight. After washing five times with TBS‐T, the membranes were incubated with horseradish peroxidase‐conjugated secondary antibody (1:50,000 dilution; Huabio) for 1 h. Western blot bands were visualized using enhanced chemiluminescence substrate (Millipore) and analyzed by Image J software.

### 2.6 Measurement of inflammatory cytokines

The cytokine concentrations in the supernatant were determined using an enzyme‐linked immunosorbent assay (ELISA) kit (Novus) according to the manufacturer's instructions. The absorbance at 450 nm was then measured using an absorbance microplate reader.

### 2.7 Immunohistochemistry

The liver and lung tissue sections were sequentially baked in a 65°C oven, deparaffinized in xylene, hydrated in grade concentrations of ethanol, dipped in the EDTA (pH: 8.0), and finally treated with 15% hydrogen peroxide to inactivate endogenous peroxidase activity. After being blocked in 5% bovine serum albumin for 1 h, the slides were incubated with the primary antibody at 4°C overnight with anti‐Nur77 (1:50 dilution), followed by a secondary antibody on the second day. After washing three times with PBS, diaminobenzidine was added as the substrate. Then, the nuclei were counterstained with hematoxylin. Finally, the slides were subjected to 1% hydrochloric acid alcohol differentiation, saturating lithium carbonate back to blue, alcohol dehydration, and xylene transparent and neutral gum seals. For each group, we randomly selected three discrete fields of vision. Image‐Pro Plus 6.0 software was used to analyze the ratio of integrated optical density to the area in each field.

### 2.8 Hematoxylin and eosin (HE) staining

For staining and histopathological score, the lung, liver, and kidney specimens were fixed in 4% formaldehyde before embedding in paraffin. Tissues were sectioned at a thickness of 3 μm and then stained with HE for light microscopic analysis. Ten nonoverlapping HE‐stained pictures were randomly selected for each group.

### 2.9 Statistical analysis

The experimental results were statistically analyzed and graphed using GraphPad Prism8. All results were reported as the mean ± SD from three independent experiments. The Student's *t*‐test was used to compare the means between the two groups. Statistical significance was evaluated using a one/two‐way analysis of variance. *p* Values were considered significant at <.05.

## 3 RESULTS

### 3.1 Dexmedetomidine reduced the production of inflammatory cytokines in LPS‐treated RAW264.7 cells

**Figure 1:**
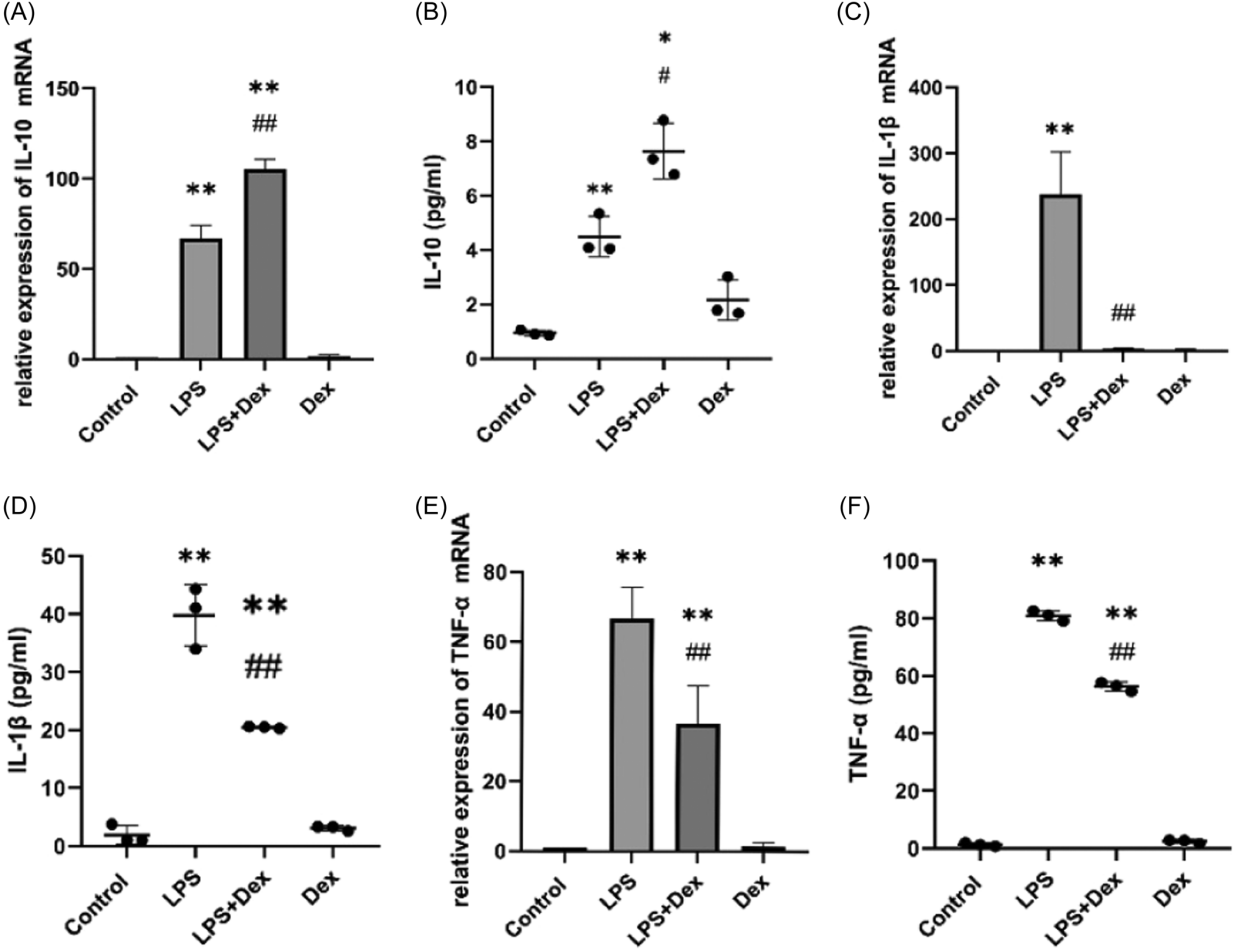
Dexmedetomidine (Dex) decreases inflammatory cytokine levels in lipopolysaccharide (LPS)‐treated RAW264.7 cells. (A) Interleukin‐10 (IL‐10) mRNA expression in LPS‐treated RAW264.7 cells. (B) IL‐10 concentrations measured by enzyme‐linked immunosorbent assay (ELISA). (C) IL‐1β mRNA expression in LPS‐treated RAW264.7 cells. (D) IL‐1β concentrations measured by ELISA. (E) Tumor necrosis factor‐α (TNF‐α) mRNA expression in LPS‐treated RAW264.7 cells. (F) TNF‐α concentrations measured by ELISA. The experiments were independently repeated in triplicate. The measurement data are expressed as mean ± SD. Statistical significance was indicated by *p < .05, **p < .01 versus the control group, and ^#^ *p* < .05, ^##^ *p* < .05 versus the LPS group.

The pathogenesis of sepsis is accompanied by the overactivation of various inflammatory factors, including IL‐1β, TNF‐α, and IL‐10. Following an infectious challenge with LPS, animals show increased circulating levels of pro‐inflammatory mediators. Neutralization of inflammatory factors can improve survival in animal models of sepsis.^31^ In this study, we investigated the relationship between Dex and Nur77 by evaluating the mRNA expressions of the inflammatory cytokines IL‐10, IL‐1β, and TNF‐α (Figure 1A,C,E). Our results show that Dex influenced the levels of IL‐10, IL‐1β, and TNF‐α mRNA. In LPS‐treated RAW264.7 cells, Dex significantly decreased the levels of IL‐1β and TNF‐α compared to the LPS group. We also used ELISA kits to examine the production of IL‐10, IL‐1β, and TNF‐α (Figure 1B,D,F), and the results were consistent with those obtained from the mRNA expression analysis. These findings suggest that dexmedetomidine can reduce the inflammatory response in RAW 264.7 cells.

### 3.2 Dexmedetomidine upregulated the expression of Nur77 in LPS‐treated RAW264.7 cells

**Figure 2:**
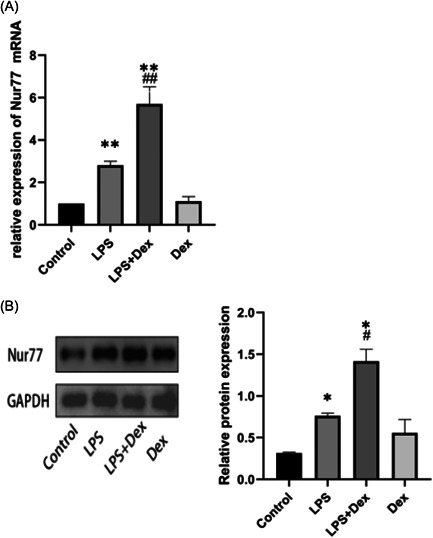
Dexmedetomidine (Dex) increases nuclear receptor 77 (Nur77) expression in lipopolysaccharide (LPS)‐treated RAW264.7 cells. (A) LPS treatment induced the expression of Nur77, and Dex upregulated Nur77 expression in LPS‐treated macrophages. The mRNA levels of Nur77 in LPS‐treated RAW264.7 cells were measured relative to the control group, and four groups of RAW264.7 cells were analyzed: control, LPS, LPS + Dex, and Dex. (B) Western blot analysis analysis of Nur77 expression in RAW264.7 cells. The experiments were independently repeated in triplicate, and the Nur77 expression data are expressed as mean ± SD. Statistical significance was indicated by *p < .05, **p < .01 versus the control group, and ^#^ *p* < .05, ^##^ *p* < .05 versus the LPS group.

We analyzed the mRNA and protein levels of Nur77 in RAW264.7 cells from different groups after treating the cells with LPS (500 ng/mL) for 3 h to investigate the impact of Dex and Nur77 on RAW264.7 cells. In the LPS group, we observed that LPS stimulation increased the expression of Nur77 mRNA in RAW264.7 cells compared to the control group. However, Dex further increased the expression of Nur77 mRNA in LPS‐treated cells (Figure 2A). Similar results were obtained for the Nur77 protein level (Figure 2B). Notably, we did not observe any significant effects of Dex alone on the expression of Nur77. These data suggested that Dex could induce the expression of Nur77 in RAW264.7 cells treated with LPS.

### 3.3 The effects of dexmedetomidine on inflammation in LPS‐treated RAW264.7 cells were mediated by Nur77

**Figure 3:**
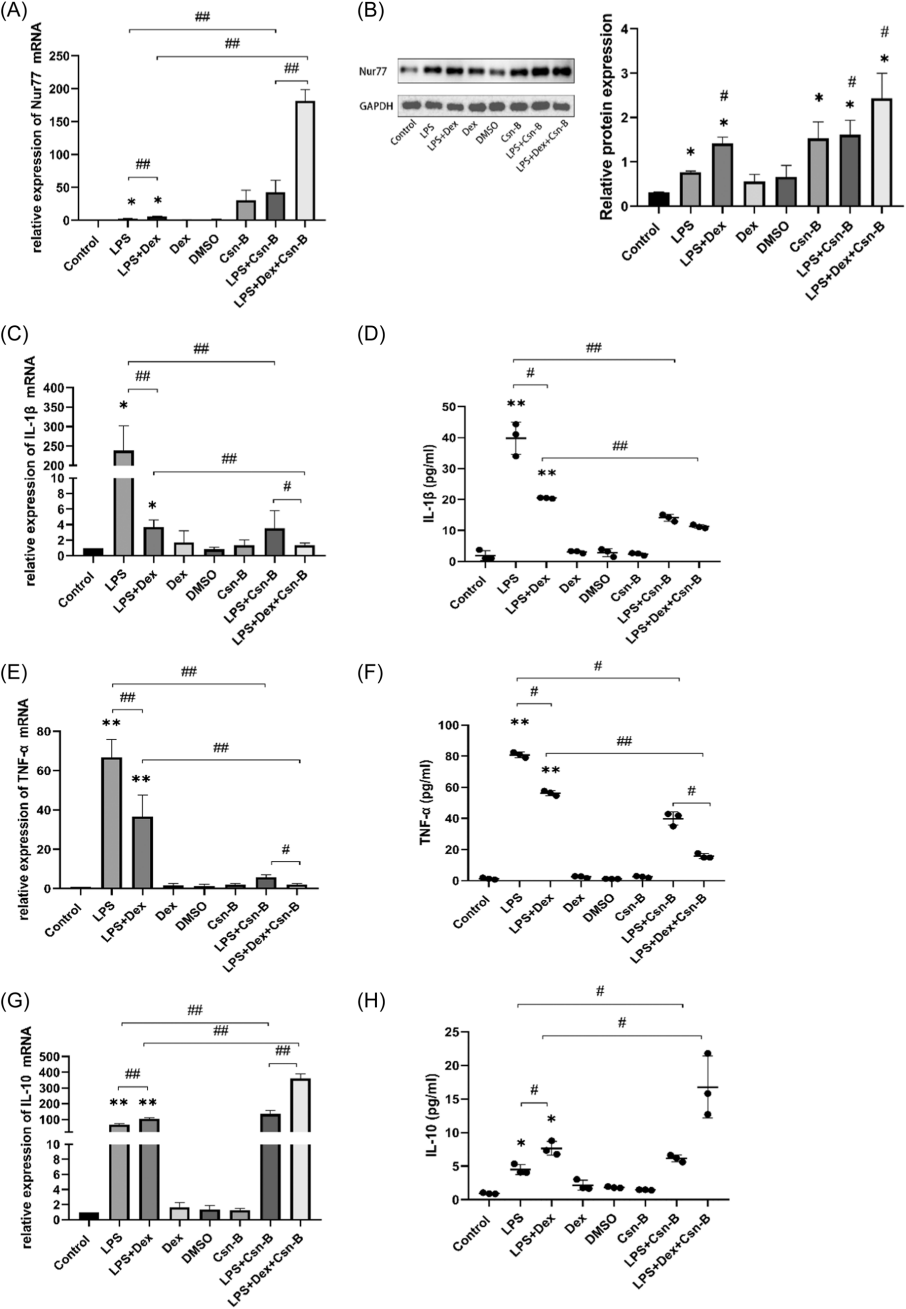
The anti‐inflammatory effect of dexmedetomidine (Dex) is similar to that of the nuclear receptor 77 (Nur77) agonist Csn‐B in lipopolysaccharide (LPS)‐treated cells. (A) The mRNA expression of Nur77 in LPS‐treated RAW264.7 cells relative to different groups treated with Csn‐B (10 mg/mL). (B) Western blot analysis analysis of Nur77 expression in RAW264.7 cells. (C) The mRNA expression of interleukin‐1β (IL‐1β) in LPS‐treated RAW264.7 cells compared to the control. (D) IL‐1β concentrations measured by enzyme‐linked immunosorbent assay (ELISA). (E) The mRNA expression of tumor necrosis factor‐α (TNF‐α) in LPS‐treated RAW264.7 cells relative to different groups treated with Csn‐B. (F) TNF‐α concentrations measured by ELISA. (G) The mRNA expression of IL‐10 in LPS‐treated RAW264.7 cells relative to different groups treated with Csn‐B. (H) IL‐10 concentrations measured by ELISA. The experiments were independently repeated in triplicate, and the inflammatory cytokine expression data are expressed as mean ± SD. Statistical significance was indicated by *p < .05, **p < .01 versus the control group, and ^#^ *p* < .05, ^##^ *p* < .05.

Treatment with LPS increased the inflammatory response of cultured cells compared to the control group. Furthermore, it was observed that overexpression of the nuclear receptor 4A1 (NR4A1) in sepsis suppressed the inflammatory response (Figure 3). In this study, we used the classical Nur77 agonist Csn‐B to induce strong expression of Nur77 before the production of inflammatory factors to understand the relationship between Dex and Nur77. Specifically, RAW264.7 cells were treated with Csn‐B and analyzed by western blot analysis and RT‐qPCR. Inflammatory mediators are upregulated during LPS‐induced sepsis. Treatment with Csn‐B and Dex alone did not alter the expression of IL‐1β and TNF‐α. However, LPS incubation significantly increased the levels of IL‐1β and TNF‐α compared to controls. Notably, this elevation was significantly reduced following treatment with Csn‐B, as shown in Figure 3. In LPS‐stimulated cells, treatment with Dex decreased the levels of IL‐1β and TNF‐α. These effects of Dex were further enhanced by treatment with Csn‐B (Figures 3C–F). Additionally, treatment with Dex increased the levels of IL‐10 (Figure 3G,H). These results suggest that dexmedetomidine may partially upregulate the expression of Nur77 and limit the production of pro‐inflammatory cytokines during sepsis.

**Figure 4:**
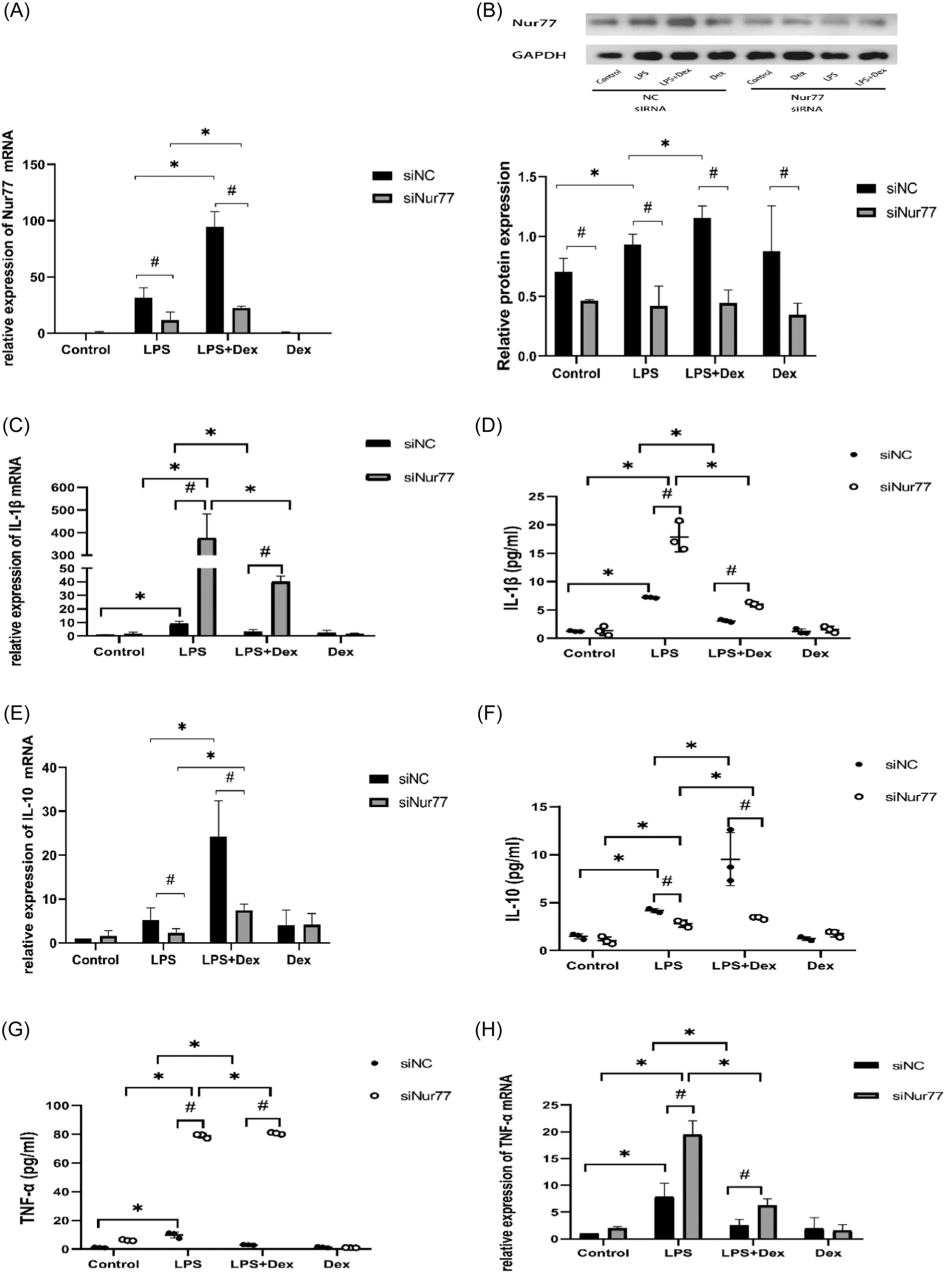
Dexmedetomidine (Dex) modulates inflammation via nuclear receptor 77 (Nur77). (A) The expression of Nur77 mRNA in lipopolysaccharide (LPS)‐treated RAW264.7 cells was evaluated. (B) Nur77 protein expression was analyzed by Western blot analysis. (C) The mRNA expression of interelukin‐1β (IL‐1β) was measured in LPS‐treated cells. (D) IL‐1β concentrations measured by enzyme‐linked immunosorbent assay (ELISA). (E) The mRNA expression of IL‐10 was measured in LPS‐treated cells. (F) IL‐10 concentrations measured by ELISA. (G) The mRNA expression of TNF‐α was measured in LPS‐treated cells. (H) TNF‐α concentrations measured by ELISA. Cells were transfected with siNC or siNur77 for 72 h, and then treated with Dex (10 μM) for 30 min before exposure to LPS. The data are expressed as mean ± SD. *p < .05, ^#^ *p* < .05.

To confirm that the anti‐inflammatory effects of Dex on LPS‐induced RAW264.7 cells were related to Nur77, we performed loss‐of‐function studies using the siRNA knockdown approach and examined the effects on the expression of inflammatory factors. We found that siRNA knockdown resulted in a significant reduction in Nur77 levels in RAW264.7 cells (Figure 4A,B). Data showed a dramatic increase in the levels of IL‐1β and TNF‐α in LPS‐induced RAW264.7 cells, and this increase was further exacerbated in Nur77 siRNA cells (Figure 4C,D). Consistently, the expression of IL‐10 and Nur77 in LPS‐stimulated RAW264.7 cells were significantly downregulated by Nur77 siRNA but not completely abolished (Figure 4E,F). Dex attenuates the levels of pro‐inflammatory factors in LPS‐treated Nur77 siRNA cells.

These results confirm that Nur77 knockdown has a significant impact on the production of inflammatory cytokines, but Dex can partially exert anti‐inflammatory effects by upregulating Nur77.

### 3.4 Dexmedetomidine alleviated CLP‐induced acute multiple organ injury

**Figure 5:**
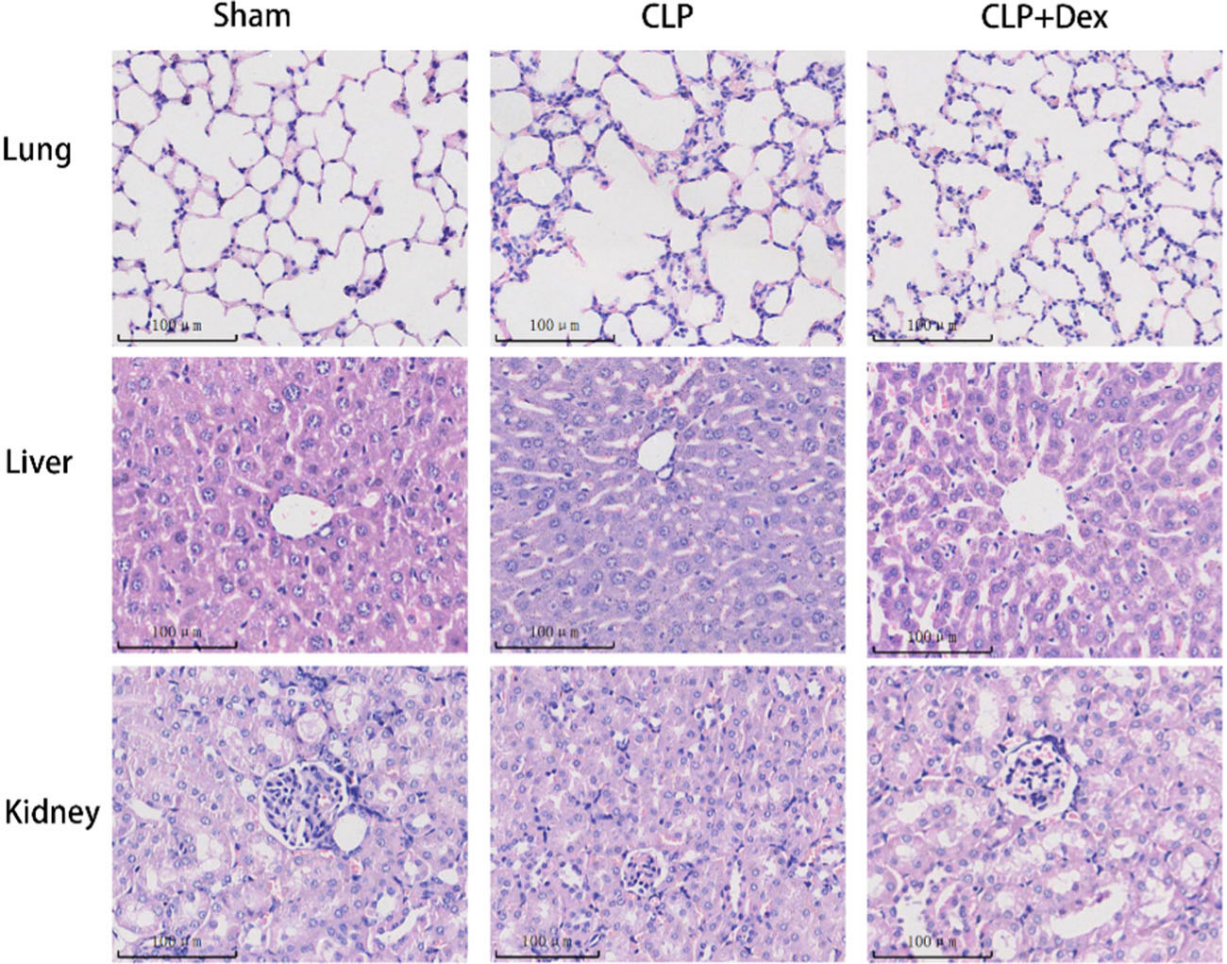
The protective effect of dexmedetomidine (Dex) against acute multiple organ injury induced by cecal ligation and puncture (CLP) was evaluated in mice. CLP mice were administered Dex at a dose of 40 μg/kg at the specified time points, and histological analysis of lung, kidney, and liver tissues was performed using hematoxylin and eosin. The sample size for each group was *n* = 9.

The study conducted histopathological evaluation of lung, liver, and kidney tissues using HE staining 3 h after CLP modeling (Figure 5). In the CLP group, lung tissue exhibited fractures and collapsed alveoli with significant inflammatory cell infiltration. The liver showed marked steatosis and mild inflammation. However, in the CLP + Dex group, lung tissue exhibited a more complete lung tissue structure with fewer inflammatory cells, and the liver showed mild steatosis only. In the CLP group, the kidney tissues exhibited more inflammatory cell infiltration and significant glomerular injury compared to the sham group, which exhibited vacuolar degeneration. In contrast, the CLP + Dex group showed significantly alleviated pathological changes in the kidney compared to the CLP group. No mice died during the experiment. Overall, these results suggest that Dex administration can reduce the severity of pathological changes in lung, liver, and kidney tissues induced by CLP.

### 3.5 Dexmedetomidine increased CLP‐induced levels of Nur77 in mice lung and liver

**Figure 6:**
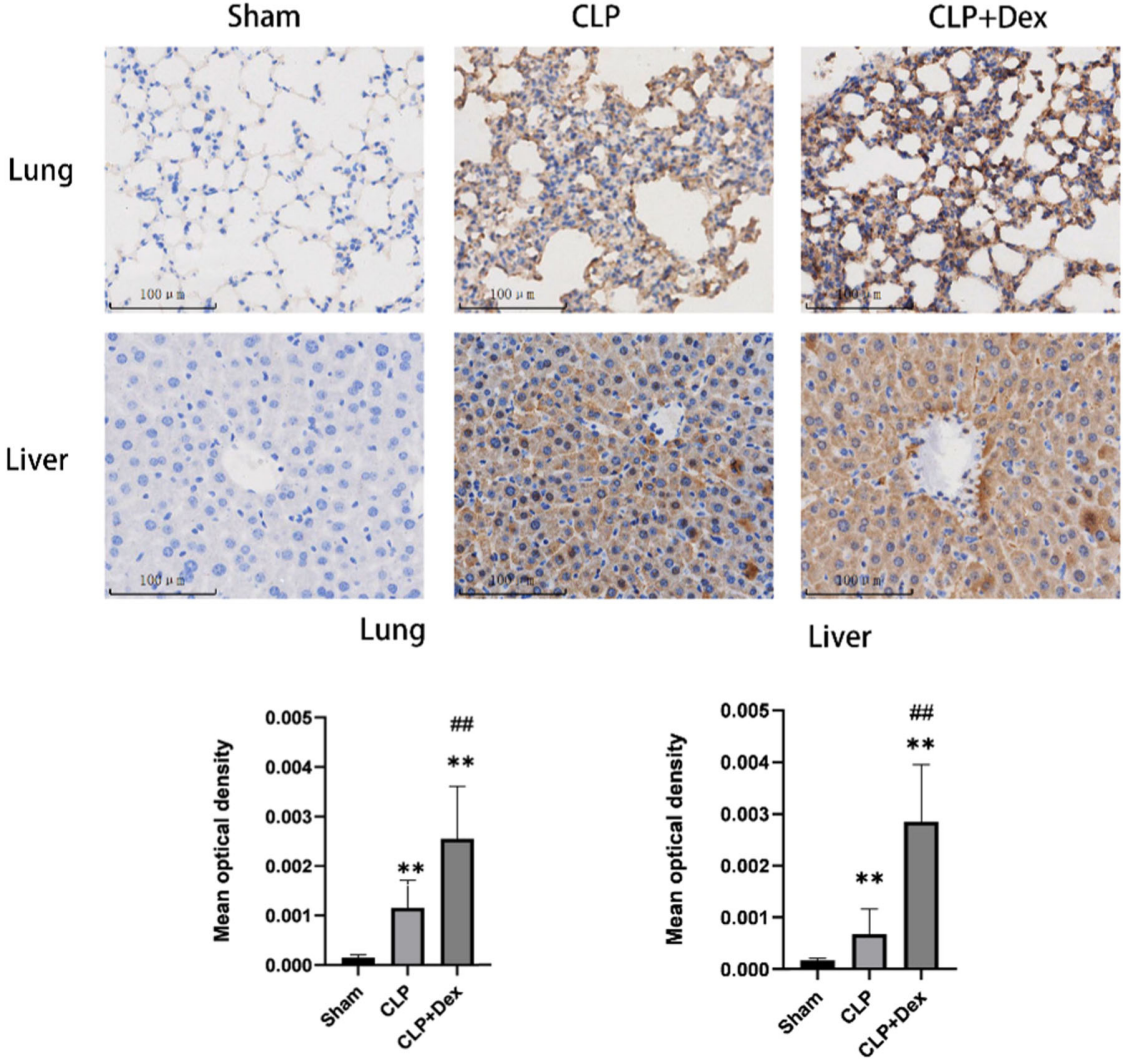
The effect of dexmedetomidine (Dex) on the expression of nuclear receptor 77 (Nur77) in cecal ligation and puncture (CLP) mice was evaluated. CLP mice were administered Dex at a dose of 40 μg/kg at the specified time points, and Nur77 protein levels in lung and liver tissues were examined 3 h after treatment using immunohistochemistry. The sample size for each group was *n* = 9. The data are presented as the mean ± SD, and statistical analysis indicated significant differences between the Sham and CLP groups (**p < .01), as well as between the CLP and Dex‐treated groups (^##^ *p* < .01).

Nur77 expression was upregulated in the mouse lung and liver after Dex administration. As shown in Figure 6, we found that protein levels of Nur77 were dramatically increased in the lung and liver relatively soon after injury, as demonstrated by a sharp increase in protein levels at 3 h after the operation. The expression of protein Nur77 was obviously decreased in the CLP group compared to the CLP + Dex group but increased significantly compared to the sham group (Figure 6). Interestingly, we did not detect any significant changes in levels of Nur77 in kidney 3 h after CLP, probably suggesting that the specific sites for Dex‐mediated regulation of Nur77 responded to early inflammatory injury.

## 4 DISCUSSION

Our study showed that Dex could attenuate inflammation and improve lung, liver, and kidney injury sepsis by partially upregulating Nur77. Sepsis is a major problem worldwide, although the risk of death from sepsis has decreased as more efforts have been invested in scientific research and clinical practice.^30^ As we know, a notable inflammatory response is the major cause of death in the early stage of sepsis.^32^ Dex, an α_2_ adrenergic receptor agonist, has sedative and pain‐killing effects. The pharmacological effects of Dex are not limited to α2 adrenergic receptors, and its protective effects can be achieved through various molecular mechanisms, including inhibition of ion channels, antioxidant properties, and inhibition of inflammatory responses.^33^, ^34^, ^35^

Dex exerts protective effects on the circulatory, nervous, and respiratory systems by affecting sodium ion channels.^36^, ^37^, ^38^ Jiang et al.^38^ found that Dex preconditioning could protect against LPS‐induced ALI by inhibiting inflammatory pathways, and the potential mechanism may be related to its effect on sodium channels. We found that Dex pretreatment reduced CLP‐induced ALI and our results are consistent with them. Some cytokines associated with inflammation, such as IL‐1β, TNF‐α, and IL‐10, have been found to play essential roles in the occurrence and development of sepsis.^39^, ^40^ It was also found that Dex reduced the production of IL‐1β and TNF‐α in LPS‐treated RAW264.7 cells. A previous study has showed that Dex could act on some inflammatory cytokines, inhibiting their production and release, including TNF‐α, IL‐6, and prostaglandins (PGs), thereby reducing inflammation and tissue damage.^35^

NR4A orphan nuclear receptors are coded by early‐response genes that are involved in the inflammatory process.^9^ Nur77 has anti‐inflammatory effects in in mice with different degrees of sepsis.^10^, ^11^, ^12^, ^13^ Nur77 can be activated by multiple stimuli, including LPS, which is widely used to induce inflammatory sepsis in both in vitro and in vivo studies.^8^ It has been shown that orphan nuclear receptor Nur77 (also known as TR3) can enhance resistance to LPS‐induced sepsis in mice by inhibiting the nuclear factor kappa B (NF‐κB) activity and suppressing aberrant cytokine production. Nur77 directly associates with p65 to block its binding to the κB element. However, this function of Nur77 is countered by the LPS‐activated p38α phosphorylation of Nur77. Therefore, dampening the interaction between Nur77 and p38α would favor Nur77 suppression of the hyperinflammatory response.^16^ Growing evidence has suggested that Dex alleviates lung intestinal ischemia–reperfusion injury and neurogenesis damage in sepsis mice through regulating the P38 MAPK signaling pathway.^41^, ^42^ Therefore, we investigated whether Dex would affect inflammatory factors and organ injury by regulating Nur77 to further clarify the anti‐inflammatory mechanism of Dex.

We successfully established an inflammatory model using LPS‐treated RAW264.7 cells. Treatment with LPS induced the upregulation of Nur77, and Dex could further increase the expression of Nur77. We found that treating LPS‐stimulated cells with Dex decreased the levels of inflammatory cytokines. However, the effects of Dex were eliminated by treatment with siRNA that knocked down Nur77. These findings suggested that the protective effect of Dex in sepsis could be partially attributed to the upregulation of Nur77.

In the CLP‐induced sepsis model, the pulmonary barrier is disrupted,^43^ and renal cortical capillary blood flow is impaired.^44^ Acute liver injury, which is a serious complication of sepsis, is closely associated with changes in inflammatory factor levels.^45^ Our study also showed that Dex could improve these organ injuries. This is consistent with another study which showed Dex alleviated liver and lung injury during sepsis and is beneficial for microcirculation in intra‐abdominal experimental sepsis models.^46^ Dex might decrease mortality and inhibit inflammation by enhancing the activity of the immune system while reducing its systemic reaction and lowering cytokine concentrations. Moreover, Dex can improve the polarization macrophage phenotype,^47^ exert a protective effect, reduce mortality, and inhibit the inflammatory response associated with endotoxic shock, the corresponding mechanisms and cellular signaling pathways remain unclear. In this study, we evaluated the relationship between Dex and Nur77. The sepsis model showed enhanced expression of pro‐inflammatory factors and organ dysfunction. However, these disorders were reversed after the upregulation of Nur77 by Dex treatment.

Several limitations should be noted in this study. First, we only assessed the early therapeutic effects of Dex in LPS‐induced cell and animal models, and we did not conduct any long‐term observations. Second, Dex has other effects, including inhibition of ion channels, antioxidation, and suppression of inflammatory responses.^36^, ^37^, ^38^ The specific mechanism by which Dex protects against sepsis‐related inflammation and organ dysfunction, which may be caused by the α‐2A effect. Furthermore, it is worthwhile to investigate whether Dex can reduce the progression of sepsis and decrease mortality by early regulation of Nur77, and whether the regulation of Nur77 and inflammatory factors by Dex is related to the NF‐κb pathway deserve further investigation.

## 5 CONCLUSION

In conclusion, this study revealed that Dex attenuated macrophage inflammation partially by upregulating Nur77 during sepsis. Furthermore, Dex effectively prevented organ injury in CLP mice. We believe that this effect of Dex is important for the treatment of sepsis and deserves further investigations by in‐depth studies to provide a more detailed mechanism.

## AUTHOR CONTRIBUTIONS

All authors made a significant contribution to the work reported, whether that is in the conception, study design, execution, acquisition of data, analysis and interpretation, or in all these areas; took part in drafting, revising or critically reviewing the article; gave final approval of the version to be published; have agreed on the journal to which the article has been submitted; and agree to be accountable for all aspects of the work.

## CONFLICT OF INTEREST STATEMENT

The authors declare no conflict of interest.

## Data Availability

The original contributions presented in the study are included in the article, further inquiries can be directed to the corresponding author.
